# β-arrestin 2在非小细胞肺癌患者血清中表达的临床意义

**DOI:** 10.3779/j.issn.1009-3419.2011.06.04

**Published:** 2011-06-20

**Authors:** 正清 吴, 文侠 童, 子辉 谭, 思愚 王, 鹏 林

**Affiliations:** 1 510060 广州，中山大学肿瘤防治中心胸科 Department of Chest Surgery, Cancer Center, Sun Yat-sen University, Guangzhou 510060, China; 2 510060 广州，华南肿瘤学国家重点实验室 State Key Laboratory of Oncology, Guangzhou 510060, China; 3 510060 广州，中山大学肿瘤防治中心妇科 Department of Gynecologic Oncology, Cancer Center, Sun Yat-sen University, Guangzhou 510060, China

**Keywords:** β-arrestin 2, 肺肿瘤, 预后因素, β-arrestin 2, Lung neoplasms, Prognostic factors

## Abstract

**背景与目的:**

非小细胞肺癌（non-small cell lung cancer, NSCLC）是肺癌中最常见的类型，具有较高的发病率及病死率。β-arrestin 2是一种介导受体脱敏的重要可溶性蛋白质，在G蛋白偶联受体介导的信号转导中具有重要调节作用。本研究旨在探讨β-arrestin 2在NSCLC患者血清中的表达，并探讨其临床意义。

**方法:**

选取留有血清标本的2005年1月-2006年12月于中山大学肿瘤防治中心治疗并确诊的NSCLC患者67例及正常体检者20例，采用ELISA方法测定选取血清标本中β-arrestin 2蛋白的表达情况，并对相应病例的临床及随访资料进行回顾性分析。

**结果:**

NSCLC患者组别均较正常人组别血清β-arrestin 2浓度低（*P* < 0.001, *P* < 0.001, *P* < 0.001）；同时Ⅰ期患者组别较Ⅲ期、Ⅳ期患者组别血清β-arrestin 2浓度高（*P* < 0.001, *P* < 0.001）；而Ⅲ期与Ⅳ期患者组别血清β-arrestin 2浓度无差异（*P*=0.273）。*Kaplan-Meier*分析显示β-arrestin 2高表达患者较中、低表达患者预后更好（*P* < 0.001, *P* < 0.001）。*COX*回归分析显示，β-arrestin 2浓度和肿瘤分期对预后具有明显意义（*P*=0.003, *P*=0.004）。

**结论:**

β-arrestin 2在正常人和NSCLC患者、不同病理分期NSCLC患者血清中浓度有差异。血清β-arrestin 2浓度影响NSCLC患者预后。

## Abstract (en)

**Background and objective:**

Non-small cell lung cancer (NSCLC) with high morbidity and mortality is the most common types of lung cancer. beta-arrestin 2 is a kind of soluble protein regulating signal transduction mediated by G protein coupling receptor. The aim of this research is to evaluate the clinical significance of β-arrestin 2 expression in the serum of NSCLC patients.

**Methods:**

The clinical and follow-up data of 20 healthy candidates and 67 patients diagnosed with NSCLC in Sun Yat-sen University Cancer Center from January 2005 to December 2006 was retrospectively analyzed. ELISA was applied to detect the expression of beta-arrestin 2.

**Results:**

The serum level of β-arrestin 2 in NSCLC patients were all significally lower than those in healthy controls (*P* < 0.001, *P* < 0.001, *P* < 0.001). The serum level of β-arrestin 2 in stage Ⅰ NSCLC patients were higher than those in stage Ⅲ as well as in stage Ⅳ (*P* < 0.001, *P* < 0.001).No statistical difference of β-arrestin 2' serum level was found between with stage Ⅲ and stage Ⅳ patients (*P*=0.273). Univariate prognostic factor analyzed by *Kaplan-Meier* method indicated patients' prognosis with high serum level of β-arrestin 2 was better than patients with low and middle (*P* < 0.001, *P* < 0.001). The serum level of β-arrestin 2 and the stage of NSCLC signally affected prognosis in *COX* regression model (*P*=0.003, *P*=0.004).

**Conclusion:**

The serum level of β-arrestin 2 had significant difffrence between NSCLC patients and healthy controls, likewise between the early and advanced NSCLC patients. The serum level of β-arrestin 2 affected NSCLC patients' prognosis.

肺癌是一种常见的恶性肿瘤^[1]^，是肿瘤相关死亡的主要原因^[2]^，近年来我国肺癌的发病率及病死率逐年增高，其中非小细胞肺癌（non-small cell lung cancer, NSCLC）约占85%^[3, 4]^。临床确诊时的病期是影响肺癌患者预后的主要因素之一^[5]^，尽管现代诊断技术已取得长足进步，但目前针对肺癌早期诊断的方法仍不尽人意，70%的患者就诊时已经是局部晚期或发生转移^[6]^，而目前肺癌患者的5年生存率仍然只有14%^[7]^。因此，寻找能早期发现和诊断肺癌或者评价其预后的肿瘤标志物已成为目前肺癌临床和基础研究的主要方向。β-arrestin 2是一类介导受体脱敏的重要可溶性蛋白质，是GPCRs信号通路的重要负调节因子，目前在乳腺癌^[8, 9]^和前列腺癌^[10]^的研究中发现它对预后预测具有一定的意义。本研究通过测定NSCLC患者血清中β-arrestin 2的表达情况，初步探讨β-arrestin 2表达与NSCLC分期及预后的关系。

## 1 材料与方法

### 1.1 一般材料

① 标本：本研究所采用的NSCLC患者治疗前的血清标本全部取自中山大学肿瘤防治中心的住院病人，所有病例均经病理组织学确诊为NSCLC入院后次日清晨空腹状态下采血，均为治疗前阶段采血。所采用的正常人血清标本均来自中山大学肿瘤防治中心体检的正常健康非肿瘤人群。血标本经离心后，保存于-80 ℃冰箱；②试剂：本研究所采用的β-arrestin 2 ELISA试剂盒采购自广州吉泰新绎生物科技公司；③仪器：SpectraMax m5多功能酶标仪（Molecular devices公司）、5814型离心机（Eppendorf公司）、多道移液器（Gilson公司）等。

### 1.2 临床基本资料

选取2005年1月-2006年12月在中山大学肿瘤防治中心就诊治疗并确诊为NSCLC的67例患者及20例正常体检者的血清标本。采集住院患者血标本已经中山大学肿瘤防治中心伦理委员会审核通过，且均告知患者采集血标本之权利及义务，并征求患者及家属同意。NSCLC患者中位年龄为58岁（36岁-75岁）；男性36例（53.7%），女性31例（46.3%）；病理证实鳞癌28例（41.8 %），腺癌39例（58.2 %）；高分化8例（12%），中分化23例（34.3%），低分化36例（53.7%）；有肿瘤家族史16例（23.9%），无肿瘤家族史51例（76.1%）；抽烟者27例（40.3%），不抽烟者40例（59.7%）。末次随访至2010年11月，随访时间1个月-68个月，中位随访时间为63个月。分期采用UICC 2002年第6版TNM分期系统，具体病理类型及分期详见表 1。

**1:** *β*-arrestin 2表达水平与临床病理因素的关系 The relations of clinical and pathologic factors with level of *β*-arrestin 2

Characteristic	*n*	Level of *β*-arrestin 2 (ng/mL, p25-p75)	*P*
Gender			0.051
Female	31	1.000 (0.199-1.358)	
Male	36	0.418(0.100-1.000)	
TNM stage			
Ⅰ	30	1.185 (1.000-1.589)	< 0.001 (Ⅰ *vs* Ⅲ)
Ⅲ	18	0.196 (0.084-0.284)	< 0.001 (Ⅰ *vs* Ⅳ)
Ⅳ	19	0.356 (0.008-0.634)	0.273 (Ⅲ *vs* Ⅳ)
Pathological types			0.173
Squamous cell carcinoma	28	1.000 (0.176-1.381)	
Adenocarcinoma	39	0.460(0.195-1.000)	
Differentiation degree			
Low	36	0.586 (0.144-1.000)	0.138 (Low *vs* Middle)
Middle	23	1.000(0.369-1.382)	0.620 (Low *vs* High)
High	8	0.452 (0.056-0.988)	0.152 (Middle *vs* High)
Family history			0.097
Yes	16	1.031 (0.426-1.587)	
No	51	0.538 (0.127-1.002)	
Smoke			0.137
Yes	27	1.000 (0.196-1.412)	
No	40	0.585 (0.145-1.000)	

### 1.3 实验方法

采用ELISA法测定肺癌及正常人群血清标本中β-arrestin 2的表达情况，并用酶标仪测定。①配制浓度为10 ng/mL、5 ng/mL、2.5 ng/mL、1.25 ng/mL、0.625 ng/mL、0.312 ng/mL、0.156 ng/mL的β-arrestin 2标准品100 μL，分别加入A1-A7标准孔中。A8-A9空白孔加样品稀释液100 μL。余检测孔每孔加待测样品各100 μL。酶标板上加膜覆盖，37 ℃温育2 h；②弃去液体，甩干，不用洗涤。待干燥后每孔加稀释抗体各100 μL。酶标板上加膜覆盖，37 ℃温育1 h。用200 μL洗涤液洗板3次，待干燥后每孔加酶标抗体各100 μL。酶标板上加膜覆盖，37 ℃温育30 min；③同上洗板5次后，每孔加底物各90 μL。酶标板上加膜覆盖，37 ℃温育约15 min，直到标准孔前3-4孔有明显的梯度蓝色。然后每孔加终止溶液各50 μL，此时蓝色立转黄色。立即用酶标仪在450 nm波长测量各孔的OD值。

### 1.4 预后评价方法

采用总生存时间进行预后评价。总生存时间为从确诊时间开始到末次随访时间或死亡时间。失访者以末次随访时间计算。

### 1.5 统计学分析

采用SPSS 13.0统计软件进行统计分析，正态分布的计量资料采用Mean±SD标准差表示，两组间比较用非配对*t*检验；偏态分布的计量资料采用中位数（P25-P75）表示，两组间比较用*Wilcoxon*秩和检验。离散型变量的比较采用χ^2^检验。生存率计算用寿命表法，预后比较采用*Kaplan-Meier*法，多因素预后分析采用*COX*回归分析。以*P* < 0.05为有统计学差异。

## 2 结果

### 2.1 实验结果

#### 2.1.1 标准曲线绘制

根据酶标仪测定各标准孔的OD值，以标准液浓度为横坐标（对数坐标），标准孔吸光度为纵坐标（对数坐标），绘制标准曲线（图 1）。相关系数*r*=0.986。

**1:**
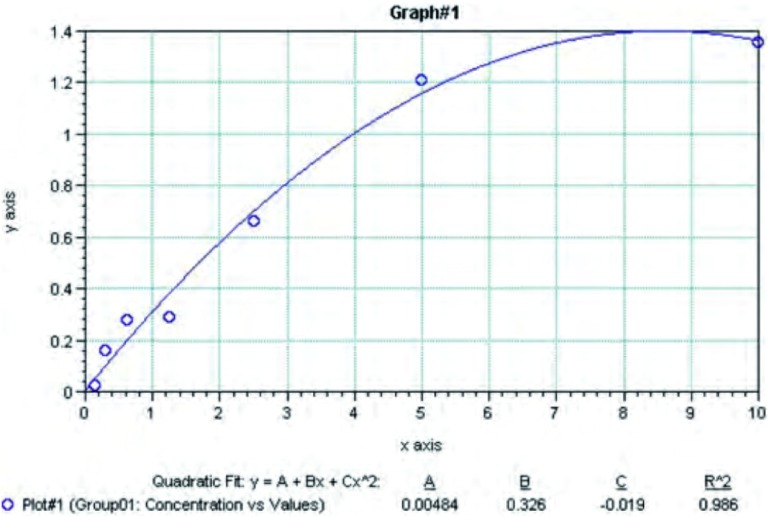
标准曲线图 The chart of standard curve

#### 2.1.2 β-arrestin 2测定结果

根据酶标仪测定各检测孔样品的OD值，计算出各孔β-ar rest in 2的实际浓度。β-arrestin 2浓度中位数为1.000 ng/mL（0.253-1.934）；其中正常人组β-ar rest in 2浓度中位数为2.047 ng/mL（2.000-2.433）；Ⅰ期患者β-arrestin 2浓度中位数为1.185 ng/mL（1.000-1.589）；Ⅲ期患者β-arrestin 2浓度中位数为0.196 ng/mL（0.084-0.284）；Ⅳ期患者β-arrestin 2浓度中位数为0.356 ng/mL（0.008-0.634）。

#### 2.1.3 β-arrestin 2与临床病理因素关系分析结果

采用*Wilcoxon*秩和检验法比较各期NSCLC患者血清β-arrestin 2浓度：Ⅰ期及Ⅲ期、Ⅳ期患者组别均较正常人组别血清β-arrestin 2浓度低（*P* < 0.001, *P* < 0.001, *P* < 0.001）；Ⅰ期患者组别较Ⅲ期、Ⅳ期患者组别血清β-arrestin 2浓度高（*P* < 0.001, *P* < 0.001），而Ⅲ期与Ⅳ期患者组别血清β-arrestin 2浓度无明显差异（*P*=0.273）。比较不同分化肿瘤程度NSCLC癌患者，高分化与中分化、低分化患者组别血清β-arrestin 2浓度均无明显差异（*P*=0.152, *P*=0.620, *P*=0.138）。比较不同病理类型、不同性别、有无家族史、是否吸烟患者组别血清β-arrestin 2浓度无明显差异（*P*=0.173, *P*=0.051, *P*=0.097, *P*=0.137）（表 1）。

### 2.2 预后分析结果

67例NSCLC患者，末次随访至2010年11月，随访时间1个月-68个月，总的5年生存率为47%。其中Ⅰ期患者的5年生存率为83%，Ⅲ期患者的5年生存率为17%，Ⅳ期患者的5年生存率为11%。

按照β-arrestin 2浓度的四份位间距将NSCLC患者分为低（0 ng/mL-0.253 ng/mL）、中（0.254 ng/mL-1.000 ng/mL）、高（>1.001 ng/mL）三组。采用*Kaplan-Meier*法比较β-arrestin 2高、中、低表达患者预后，高表达患者较中、低表达患者预后更好（*P* < 0.001, *P* < 0.001），生存曲线见图 2。将肿瘤分期、β-arrestin 2浓度、病理类型、肿瘤分化程度、性别纳入*COX*回归，*COX*回归分析显示β-arrestin 2浓度和肿瘤分期对NSCLC患者预后具有统计学意义（*P*=0.003, *P*=0.004）（表 2）。

**2:**
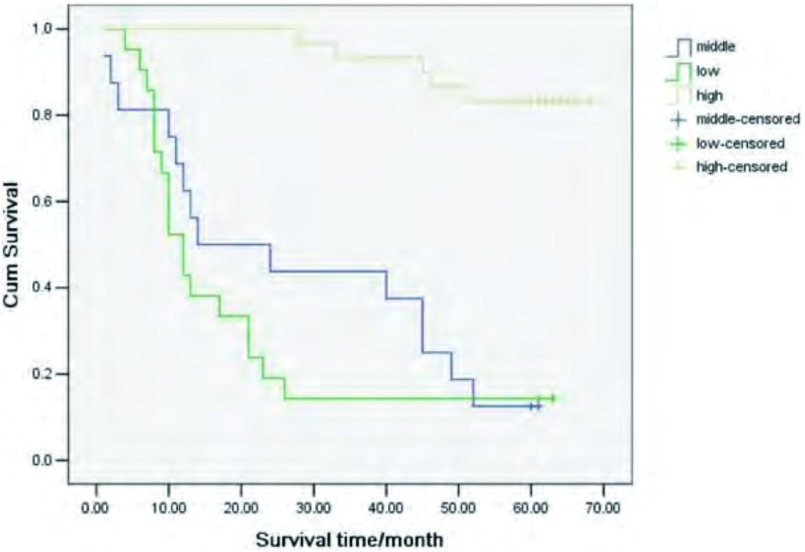
不同*β*-arrestin 2表达患者生存曲线图 The survive curves of patients with different level of *β*-arrestin 2

**2:** 多因素分析临床病理学因素对总生存时间的影响 The influence of clinical and pathologic factors to total survival time analysed by multiariate *COX* regression

Prognostic factors	B	SE	Wald	df	*P*	Exp(B)
Level of *β*-arrestin 2	-0.963	0.320	9.084	1	0.003	0.714
Stages	0.897	0.315	8.082	1	0.004	4.550
Exp(B): hazard ratio.						

## 3 讨论

人β-arrestin 2是一类介导受体脱敏的可溶性蛋白质，是GPCRs信号通路的重要负调节因子，与GPK联合作用，可以使GPCRs对激动剂的敏感性下降，发生受体的脱敏反应，从而调节受体内吞、信号转导及细胞凋亡等^[11]^。Wang等^[12]^报道了β-arrestin 2高表达可以减少Mdm2引起的p53降解，从而提高了p53介导的细胞凋亡。目前在前列腺癌和乳腺癌的研究中发现β-arrestin 2呈降低趋势，具有一定的预后预测意义。目前尚未有β-arrestin 2在人类肺癌中的相关报道，我们就本研究的结果初步探讨β-arrestin 2表达与NSCLC分期及预后的关系。

Raghuwanshi等^[13]^报道在鼠肺癌模型中，β-arrestin 2起到抑制肿瘤生长及转移的作用。本研究中，β-arrestin 2在肿瘤患者组血清中明显降低，同时在晚期患者组浓度又较早期患者组明显降低。因此，β-arrestin 2有可能在NSCLC患者的早期诊断中发挥作用，并可能有助于肿瘤分期的预判。但进一步的验证有待于大样本量的研究。

本研究中，β-arrestin 2在不同分化程度（高分化、中分化、低分化）NSCLC患者血清中表达无差异（*P*=0.152, *P*=0.620, *P*=0.138），不同病理类型、不同性别、有无家族史、是否吸烟患者组别血清β-arrestin 2浓度亦无明显差异（*P*=0.173, *P*=0.051, *P*=0.097, *P*=0.137）。因此，β-arrestin 2可能与上述临床病理因素无明显关系。

本组67例NSCLC患者，总的5年生存率为47%，其中Ⅰ期、Ⅲ期、Ⅳ期5年生存率分别为83%、17%、11%。与Pfannschmidt ^[14]^、刘树库^[15]^等报道基本相符。关于β-arrestin 2表达与NSCLC患者预后关系的报道较少，本研究中，*Kaplan-Meier*法比较β-arrestin 2高、中、低表达患者预后，高表达患者较中、低表达患者预后更好（*P* < 0.001, *P* < 0.001）。同时*COX*回归分析显示，β-arrestin 2浓度对预后具有明显意义。因此，β-arrestin 2有可能有助于肿瘤患者预后的判断，但也有待于进一步大样本量试验的验证。

综上所述，血清β-arrestin 2浓度在正常人和NSCLC患者、不同病理分期NSCLC患者血清中有明显浓度差异。*Kaplan-Meier*预后比较和*COX*回归分析显示β-arrestin 2对预后具有明显意义。因此β-arrestin 2可能在对NSCLC患者的早期诊断、预判肿瘤分期、预判预后等方面发挥作用。
